# CD73 Is Dispensable for the Regulation of Inflationary CD8^+^ T-Cells after Murine Cytomegalovirus Infection and Adenovirus Immunisation

**DOI:** 10.1371/journal.pone.0114323

**Published:** 2014-12-09

**Authors:** Stuart Sims, Julia Colston, Vince Emery, Paul Klenerman

**Affiliations:** 1 Peter Medawar Building for Pathogen Research, Nuffield Department of Medicine, University of Oxford, Oxford, United Kingdom; 2 Department of Microbial and Cellular Sciences, University of Surrey, Guildford, Surrey, United Kingdom; University of Liverpool, United Kingdom

## Abstract

The ecto-5'-nucleotidase (CD73) is expressed by T-cell subsets, myeloid derived suppressive cells and endothelial cells. It works in conjunction with CD39 to regulate the formation and degradation of adenosine *in vivo*. Adenosine has previously been shown to suppress the proliferation and cytokine secretion of T-cells and recent evidence suggests that inhibition of CD73 has the potential to enhance T-cell directed therapies. Here we utilised a CD73 knockout mouse model to assess the suppressive ability of CD73 on CD8^+^ T-cell classical memory and memory “inflation”, induced by murine cytomegalovirus (MCMV) infection and adenovirus immunisation. We show that CD73 is dispensable for normal CD8^+^ T-cell differentiation and function in both models. Thus CD73 as a suppressor of CD8^+^ T-cells is unlikely to play a deterministic role in the generation and functional characteristics of antiviral memory in these settings.

## Introduction

Cytomegalovirus (CMV) is a herpesvirus that following initial infection has the capacity to enter latency and establish a persistent infection. In both human and murine systems the virus is well controlled by the immune system, however, controlling reactivation requires consistent T-cell immune surveillance [1]. Virus-specific CD8^+^ T-cells do not uniformly contract after CMV enters latency and some are maintained at a high frequency, with some single epitope-specific CD8^+^ T-cells making up to 10% of the CD8^+^ T-cell compartment in both mice and human systems: these are functional and have an effector memory phenotype [2], [3]. Recent studies have shown that CMV is an effective vaccine vehicle due to its ability to induce high numbers of functional effector CD8^+^ T-cells that traffic to peripheral tissue [4], [5].

Memory inflation was first described in MCMV-infected BALB/c mice [6] and further analyzed in longitudinal studies [2]. During acute infection of C57BL/6 mice there are CD8^+^ T-cells specific for at least 24 MCMV epitopes derived from 18 viral proteins. However, in latency, all but five epitope specific CD8^+^ T-cells contract [7]. These inflationary responses are derived from both immediate early and late genes; the general assumption is that these populations are the direct result of on-going immune activation driven by viral reactivation.

Inflationary T-cells produce perforin, granzymes, can kill target cells, and they secrete IFNγ and TNFα but produce limited IL-2 and have a phenotype of repeated antigen stimulation, displaying an effector memory phenotype (CD62L^Lo^, CD127^Lo^, CD27^Lo^, KLRG1^Hi^) [3]. In contrast, T-cells that were immunodominant during the acute phase, and contracted in the memory phase, persist at low levels and have a central memory phenotype (CD62L^Hi^, CD127^Hi^, CD27^Hi^, KLRG1^Lo^). Inflationary MCMV T-cells express low levels of IL-7 and IL-15 receptors, cytokines important in homeostatic maintenance of memory T-cells. In humans inflationary HCMV T-cells have short telomeres, indicating extensive proliferation [8]. The frequency of these cells correlates with the amount of CMV virus at the peak of infection [9]. Importantly they remain functional and do not show features of exhaustion [3], [10]. The specific combinations of stimuli that produce these effector cells and induce proliferation are poorly understood, although progress is being made in this area [11]–[13].

Memory inflation is not limited to MCMV, and has subsequently been observed in responses to other persistent infections. With HSV1, CD8^+^ T-cells specific for glycoprotein B-derived epitope gradually accumulate [14]. These gB-specific CD8^+^ T-cells block HSV-1 reactivation from latently infected ganglia [15], remain functional and are maintained at a high frequency for the lifetime of the host. Memory inflation also occurs in a murine polyomavirus (PyV) persistent infection model. CD8^+^ T-cells recognising a peptide presented by Q9, an MHC class Ib molecule, increase in frequency over 3 months before being stability maintained [16]. Additionally, inflation is not restricted to persistent infections; a replication deficient adenovirus expressing β-galactosidase has been shown to induce this CD8^+^ T-cell inflationary response [17].

A cell surface molecule of increasing interest in the context of inflammatory responses is the ecto-5'-nuceleotidase (CD73). This is a glycosyl phosphatidylinositol (GPI)-linked membrane bound glycoprotein, expressed by both immune and endothelial cells which plays an important role in maintaining barrier function, cardio-protection, ion transport and immune regulation - the latter mediated through its role in degradation of ATP (represented in Figure S1) [18]. The metabolism of ATP into its metabolites ADP, AMP and adenosine is a tightly regulated process with conversion of ATP into AMP by CD39 (NTPDase1) [19] followed by production of adenosine from AMP by CD73. Adenosine suppresses the immune response through the activation of G-protein coupled receptors, expressed on a variety of immune cells including T-cells, NK-cells, NKT-cells, macrophages, DCs, neutrophils and B-cells.

The effects of free adenosine are wide ranging. T-cells, which may express A_2A_, A_2B_ and A_3_ receptors, up-regulate adenosine receptors upon TCR stimulation and stimulation of these receptors by adenosine results in inhibition of proliferation, cytotoxicity and pro-inflammatory cytokine production [20], [21]. Stimulation of adenosine receptors on APCs induces the differentiation of alternatively activated macrophages (AAMs). These macrophages secrete less IL-2 and TNFα but increased amounts of IL-10. On DCs, adenosine inhibits maturation and proinflammatory cytokine production, impairing their ability to induce T_H1_ responses [22], [23].

The role of CD73 expression and CMV in human common variable immunodeficiency (CVID) has recently been studied [24]. Patients with CVID have defective antibody production and increased susceptibility to infection, but also show an inflammatory phenotype. Organ specific inflammation in these patients correlated with an elevated level of CMV-specific CD8^+^ T-cells, which also had a CD73^Lo^ phenotype in patients with inflammation compared to non-inflammatory CVID controls. As a consequence it was postulated that the inflammation seen in patients was a likely effect of CMV infection resulting in inflationary CD8^+^ T-cells, with a low level of CD73 expression leading to defective regulation and further contributing to an inflammatory environment.

To study the effect of CD73 on inflationary and classical memory CD8^+^ T-cells and overall control of virus replication, we analysed the frequency and phenotype of antiviral CD8^+^ T-cells in CD73 knockout mice [25] in comparison to C57BL/6 mice after MCMV infection. We did not observe an impact of CD73 knockout in this model or in the related model of adenovirus-induced memory inflation. The data suggest that CD73 expression is dispensable for induction and maintenance of immunologic memory in these settings, and that CD73-dependent generation of adenosine does not likely play a major role in the regulation of such memory pools.

## Materials and Methods

### Ethics statement

Mouse experiments were performed according to UK Home Office regulations (project licence number PPL 30/2235 and 30/2744) and after review and approval by the local ethical review board at the University of Oxford.

### Mice

C57BL/6 and CD73^−/−^ mice were bred in a specific pathogen free animal facility at University of Oxford, Biomedical Sciences (BMS), UK, or obtained from Harlan UK. The CD73^−/−^ mice were originally provided from the laboratory of Dr Linda Thompson, Oklahoma Medical Foundation. Experiments were performed using age and sex matched mice.

### Viruses

MCMV strain (Strain Smith, ATCC: VR194) was used and kindly provided by Professor U.H. Koszinoswki, Department of Virology, Max von Pettenkofer Institute, Munich Germany. MCMV was propagated and titrated on NIH 3T3 cells (ECACC, UK), stored at -80°C and injected intravenously at a dose of 1×10^6^ pfu.

### Peptides

Peptides derived from MCMV, M38_316-323_ (SSPPMFRV), M45_985-993_ (HGIRNASFI), βgal_96-103_ (DAPIYTNV) and βgal_497-504_ (ICPMYARV) and were purchased from Proimmune, Oxford, UK.

### Antibodies

Antibodies were obtained from eBioscience (San Diego, USA), BD Bioscience (Oxford, UK), Biolegend (San Diego, USA), and R&D (Abingdon, United Kingdom).

### Peptide stimulation and intracellular staining

For peptide stimulation, 1×10^6^ peripheral blood leukocytes were stimulated for 2 hours at 37°C with either 10^−4^ M M38 or 10^−4^ M M45 peptide. As a positive control cells were stimulated with phorbol myristate acetate (PMA) (50 ng/ml) and ionomycin (500 ng/ml) or left untreated as a negative control. After 2 hours GolgiPlug (1 µl/1 ml final concentration) BD Bioscience (Oxford, UK) was added to each well and cells incubated for a further 4 hours at 37°C.

Intracellular staining was carried out by fixing and permeabilising the cells using the FOXP3 Fixation/Permeabilisation Kit (eBioscience). Cells were resuspended in permeablisation buffer containing the appropriate amount of antibody and incubated for 30 minutes at 4°C. Samples were acquired on a LSRII (BD) and analysed with FlowJo software (Tree Star, USA).

### Isolation of BM, liver and lung lymphocytes

Bone marrow was isolated by washing the femur shaft with PBS. Cells were passed through a 70 µm nylon filter (BD) and red cell lysis was performed with ACK buffer. Livers were perfused with PBS then passed through a 70 µm nylon filter (BD) and lymphocytes purified by Percoll (GE healthcare) gradient centrifugation. Lungs were minced with razor blades and incubated in PBS containing 60 U/ml DNase (AppliChem) and 17 0U/ml collagenase II (Gibco) at 37°C for 45 min. Cell aggregates were dispersed by passing the digest through a 70 µm nylon filter (BD). Absolute cell counts were determined by counting viable leukocytes in an improved Neubauer chamber using Trypan blue exclusion.

### Construction of tetrameric MHC class I peptide complexes

MHC class I monomers complexed with M38 (H-2Kb) or M45 (H-2Db) peptides were produced as previously described [26] and tetramerized by addition of streptavidin–PE (BD Bioscience, Oxford, UK) or streptavidin-APC (Invitrogen, Paisley, UK) (7). The βgal (H-2 Kb) tetramers were kindly provided by the NIH tetramer core facility, Emory University, USA. Aliquots of 1×10^6^ cells or 100 µL of whole blood were stained using 50 µL of a solution containing tetrameric class I-peptide complexes (final conc. 10ηg/µl). Staining was performed at 37°C for 20 min followed by staining with mAbs at 4°C for 20 min.

### Flow cytometry

Single cell suspensions were generated from the indicated organs (see above) and 1×10^6^ cells were incubated with the indicated mAb at 4°C for 20 min; then erythrocytes were lysed with RBC Lysing Solution (BD PharMingen). Cells were analyzed using a LSRII (BD) flow cytometer and FlowJo software, gated on viable leukocytes using the live/dead fixable near-IR dead cell stain kit from Invitrogen (Paisley, UK).

### MCMV load assay

Tissues were homogenised using the MagNa Lyser instrument (Roche) and DNA was isolated using the High Pure PCR Template Preparation Kit (Roche) as per the manufactures instructions. Quantitative real-time PCR was performed using a Light cycler 480 Real-Time PCR System (Roche Diagnostics) and the LightCycler 480 probes master reaction mix (Roche Diagnostics) following the manufacturer's protocol. Data analysis was performed with LightCycler 480 Software (Roche Diagnostics). Oligonucleotides were purchased from Eurofins MWG Operon (Ebersberg, Germany). The Oligonucleotide sequences used as primers for quantitative real-time PCR were MCMV1 5-gctctattgatactccgcgcgtta-3 and MCMV2 5-aacagctagacgacagccaacgcaccg-3 (targeting MCMV gB). Thermal cycling started with HotStartTaq activation during 10 min at 95°C. Thereafter 45 cycles of amplification were run consisting of 10 s at 95°C, 30 s 60°C, and 20 s of 72°C. A negative control, containing reagents only, and serial dilutions of plasmid containing gB sequence were included in each run to generate a standard curve. Each sample was measured as a triplicate and the average concentration was used. Relative expression of samples from naive and MCMV-infected or naïve mice was calculated by the comparative cycling threshold method (ΔΔCT).

### Adenoviral vector

Recombinant adenovirus expressing the βgal protein under the control of the human CMV promoter (Ad-LacZ) and lacking E1 and E3 genes was used (19). Ad-LacZ was propagated on permissive HER-911 cells and was purified with the Vivapure AdenoPack 20 (Sartorius, Stedim biotech, 13781 Aubagne Cedex, France) according to the manufacturer's specifications. Virus titer was determined in a cytopathic effect assay. Ad-LacZ was stored at −80°C in PBS and injected intravenously at a dose of 2×10^9^ pfu.

## Results

### CD73 is differentially expressed on virus-specific CD8^+^ T-cells

**Figure 1:**
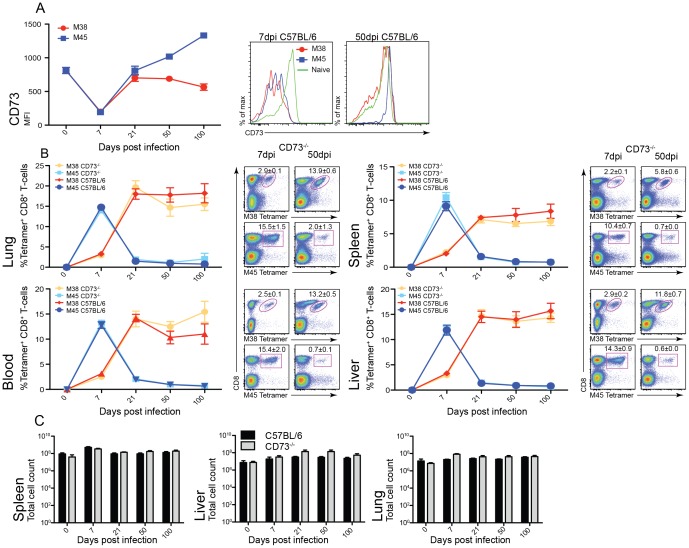
CD73 has no effect on memory inflation. C57BL/6 and CD73^−/−^ mice were infected intravenously (i.v.) with 1×10^6^ pfu MCMV and expansion of M38- (red/orange) and M45- (blue/light blue) specific CD8^+^ T-cells measured (n = 6, ±SEM). (A) Time course showing the expression levels CD73 on viral specific CD8^+^ T-cells in C57BL/6 mice from the spleen, day 0 staining is on total CD8^+^ T-cells. Histograms show representative CD73 staining on M38- (red) and M45- (blue) specific CD8^+^ T-cells at 7 and 50 days post infection in comparison to total CD8^+^ T-cells from naive mice (green). (B) Longitudinal analysis of the percentage of M38- and M45-specific CD8+ T-cells in the spleen, liver, lung and blood in naïve and at 7, 21, 50 and 100 days post infection mice. Representative flow cytometry plots of M38 and M45 tetramer staining in the spleen, liver, lung and blood at 7 and 50 days post infection in CD73^−/−^ mice. (C) Total number of lymphocytes from the spleen, liver and lung calculated.

After acute infection of C57BL/6 mice with MCMV, activated virus-specific CD8^+^ T-cells down-regulated the expression of CD73 (Figure 1.A). However, after 7 days post infection virus-specific CD8^+^ T-cells re-expressed CD73 and at 21 days post infection epitope specific CD8^+^ T-cells began to diverge in expression level, with M45-specific CD8^+^ T-cells expressing high levels of CD73 and the inflationary M38-specific CD8^+^ T-cells reaching similar levels of expression to that of naïve CD8^+^ T-cells.

Further analysis was carried out on the CD73^Hi^ and CD73^Lo^ populations within M38- and M45- specific CD8^+^ T-cells, in acute and chronic infection (day 7 and 140 post infection) and is shown in Figure S2. No difference was seen in the expression of either CD27 or CD62L between CD73^Hi^ and CD73^Lo^ populations. However, there was a decrease in CD127 expression in CD73^Lo^ compared to CD73^Hi^ at 7 and 140 days post infection with M38-specific CD8^+^ T-cells. At later time points there were too few cells within the CD73^Lo^ population of M45-specific CD8^+^ T-cells to perform comparison with the CD73^Hi^ population.

### Analysis of virus-specific CD8^+^ T-cells in CD73^−/−^ mice

To determine whether CD73 deficiency influences virus specific CD8^+^ T-cell induction and maintenance, CD73^−/−^ mice were infected with MCMV. Infection of CD73^−/−^ mice generated a normal MCMV-specific CD8^+^ T-cell response. Epitope-specific CD8^+^ T-cells were tracked using MHC Class I tetramers refolded with the M38 and M45 epitopes and lymphocytes harvested from the spleen, liver, lung and blood were analysed (Figure 1.B). M45-specific CD8^+^ T-cells showed similar responses in both CD73^−/−^ and C57BL/6, peaking at 7 days post infection with a frequency of 10–15% of CD8^+^ T-cells; this was followed by a rapid decline to a stable level of 0.6–2% of CD8^+^ T-cells that was maintained over time.

The inflationary M38-specific CD8^+^ T-cells showed no difference between CD73^−/−^ and WT infected mice, reaching a frequency of 5–15% of CD8^+^ T-cells at 21 days post infection, which was maintained into chronic infection. At 100 days post infection, there was no significant difference between C57BL/6 and CD73^−/−^ mice.

The percentage of epitope-specific CD8^+^ T-cells and the total number of lymphocytes were indistinguishable between WT and CD73^−/−^ mice (Figure 1.C). Moreover there was no change in the absolute number of virus-specific CD8^+^ T-cells.

### CD73 has no effect on proliferation or effector function of CD8^+^ T-cells

**Figure 2:**
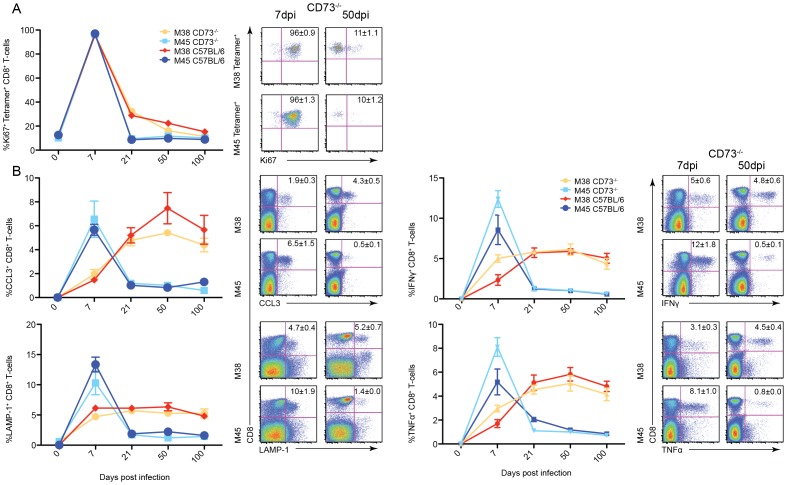
Following the proliferative and functional state of specific CD8^+^ T-cells in CD73^−/−^ mice after MCMV infection. Time courses showing the percentage of M38- (red/orange) and M45-specific CD8^+^ T-cells (blue/light blue), in the spleen on 0, 7, 21, 50 and 100 days post infection (n = 6, mean±SEM) and the shown stain (A) ICS staining for the proliferation marker Ki67. (B) Percentage CD8^+^ T-cells from the spleen producing effector molecules after stimulation with either the M38 or M45 peptide.

To analyse the proliferation kinetics of MCMV-specific CD8^+^ T-cells in CD73^−/−^ mice, intracellular staining for the proliferation marker Ki67 was performed (Figure 2.A). Both M38- and M45-specific CD8^+^ T-cells showed a similar profile in the C57BL/6 and CD73^−/−^ mice. At 7 days post infection nearly 100% of specific CD8^+^ T-cells expressed Ki67, and therefore were proliferating. M45-specifc CD8^+^ T-cells expressing Ki67 decreased to 10% at 21 days post infection and this level of expression was maintained in the memory phase. However, 30% of M38-specfic CD8^+^ T-cells still expressed Ki67 at 21 days post infection, which then gradually decreased to 15% at 100 days post infection. There was no significant difference at any time point post infection between C57BL/6 and CD73^−/−^ mice and proliferation of CD8^+^ T-cells.

It has been shown that adenosine reduces effector functions of T-cells. Therefore, we analysed production of TNFα and IFNγ in cells derived from CD73^−/−^ mice. Splenocytes were stimulated with either M38 or M45 peptide and stained for LAMP-1 and an intracellular stain was performed for CCL3, TNFα and IFNγ (Figure 2.B). There was no difference in the production of CCL3, TNFα and IFNγ or the degranulation marker LAMP-1 between MCMV peptide-stimulated cells in C57BL/6 or CD73^−/−^ mice in the spleen with the secretion profile following that of the tetramer staining (see Figure 1).

### Lack of CD73 has no effect on the differentiation of viral specific CD8^+^ T-cells

**Figure 3:**
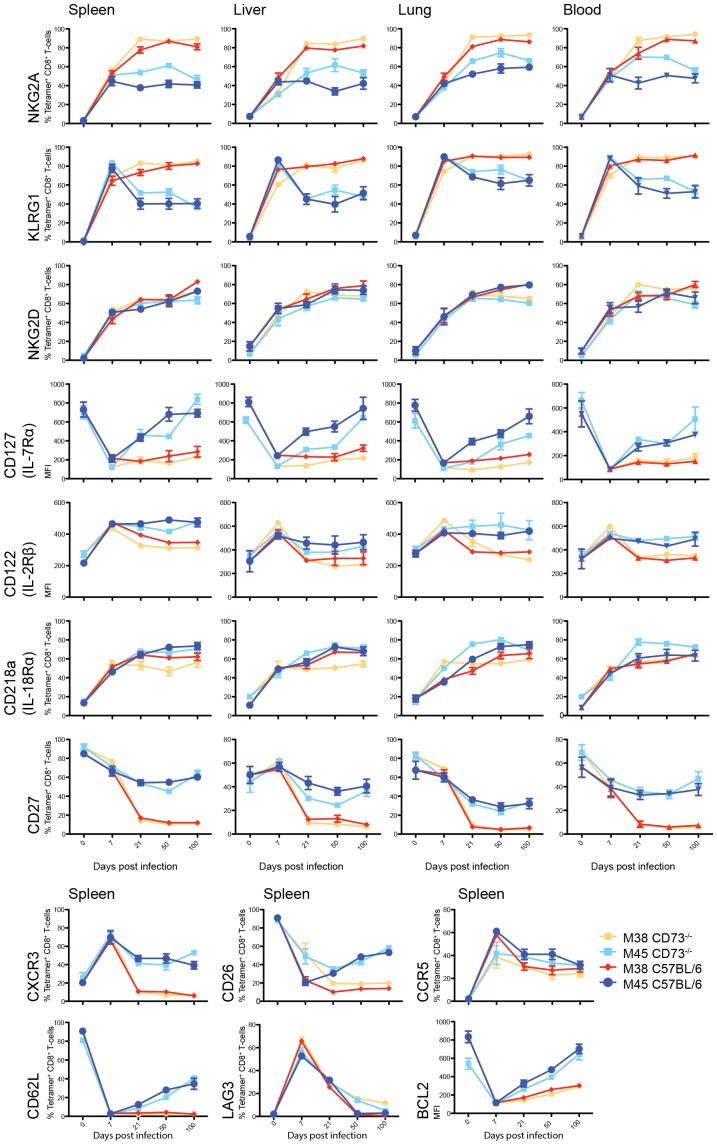
Phenotype of specific CD8^+^ T-cells in CD73^−/−^ mice after MCMV infection. Time course showing the percentage of cells expressing or MFI of the indicated markers on M38- (red/orange) and M45-specific CD8^+^ T-cells (blue/light blue), in the spleen, liver, lung and blood in comparison with naive CD8^+^ T-cells (n = 6, mean±SEM).

The phenotype of viral specific CD8^+^ T-cells gives us an insight into the signals received over time and their activation state. Again, there were no significant differences between C57BL/6 and CD73^−/−^ mice in the generation of effector and memory MCMV specific CD8^+^ T-cells (Figure 3). Lymphocytes were stained for M38- and M45-specific CD8^+^ T-cells and KLR receptors (NKG2A, KLRG1, NKG2D). M38-specific CD8^+^ T-cells maintained high expression of KLR receptors in CD73^−/−^ mice. Furthermore, the expression of the cytokine receptors CD122 and CD127, costimulatory receptor CD27 and homing receptors CXCR3, CCR5 and CD62L show that effector and memory formation in CD73^−/−^ was not influenced by the expression of CD73. Analysis of the exhaustion marker LAG3, and anti-apoptotic marker BCL-2, confirmed that CD8^+^ T-cells in CD73^−/−^ mice were not exhausted. Specific CD8^+^ T-cells in the spleen, liver, lung and blood showed the same expression profile in both C57BL/6 and CD73^−/−^ mice, suggesting that there was no reduction in the activation of virus-specific CD8^+^ T-cells and remarkably their phenotype was exactly the same in both C57BL/6 and CD73^−/−^ mice infected with MCMV.

### Analysis of the influence of CD73 depletion on the long-term control of viral loads

**Figure 4:**
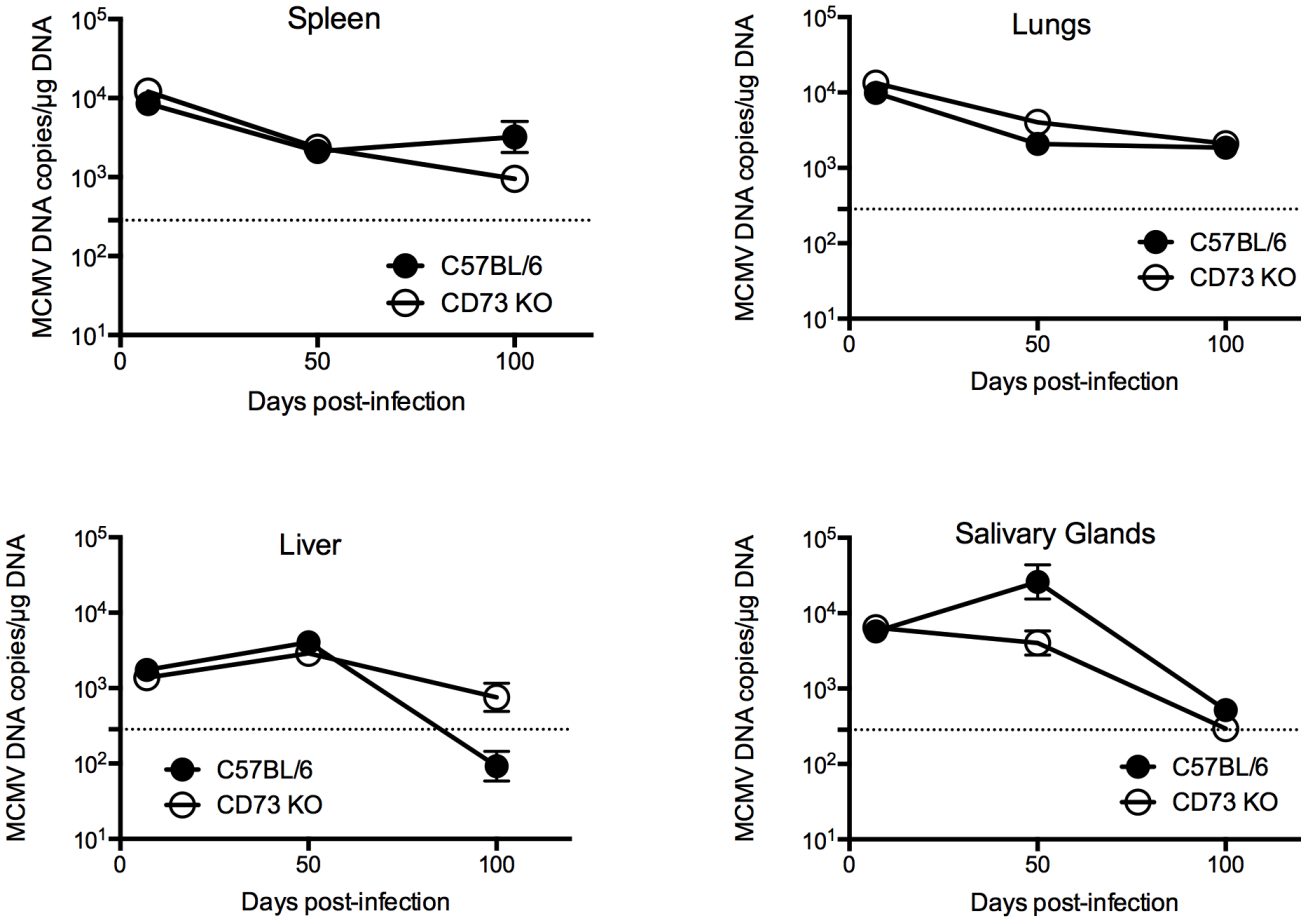
MCMV load in CD73^−/−^ mice. Time course showing MCMV viral loads in CD73^−/−^ and C57BL/6 mice 7, 21, 50 and 100 dpi. Viral load analysis performed by qRT-PCR (n = 3, mean±SEM).

CD73 is expressed on many cell types of the immune system and the *in vivo* levels of adenosine may alter the antiviral potential of CD8^+^ T-cells. Quantitative RT-PCR was used to determine the viral load in both C57BL/6 and CD73^−/−^ mice over a time course of 100 days (Figure 4). In C57BL/6 infected mice over the course of 100 days, MCMV load in the salivary glands, spleen, liver and lung declined from the peak of acute replication and similar kinetics were observed in the CD73 knockout animals (p = n.s.). In the salivary glands at day 50 post infection, the viral load is decreased in CD73^−/−^ compared to C57BL/6 by 1 log, therefore CD73 may influence protective immunity in certain sites of infection at certain times but in line with the absence of a major effect on CD8 T-cell responses in the periphery, CD73 does not play a critical role in influencing systemic infection.

### Inflationary CD8^+^ T-cells induced by Ad5-LacZ are not modulated by the lack of CD73

Inflation has recently been described using a replication-deficient adenovirus (hAd5) vector encoding the lacZ gene (17). It was shown that two epitopes from the lacZ gene elicited similar responses to MCMV-induced M38 and M45, with βgal_96_ (D8V) showing an inflationary response and βgal_497_ (I8V) representing a classical CD8^+^ T-cell response.

**Figure 5:**
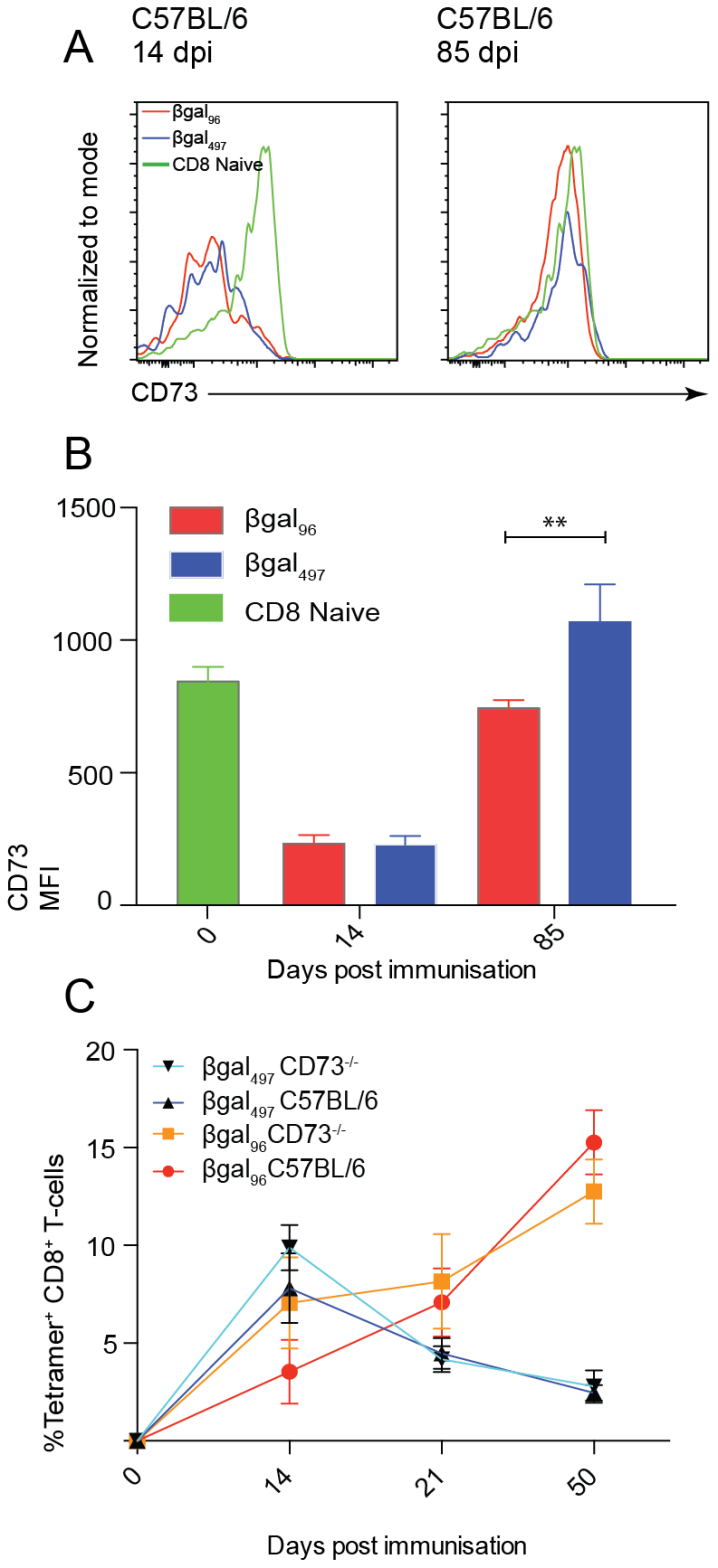
CD73^−/−^ does not affect memory inflation of CD8^+^ T-cells induced by Adeno-LacZ. CD73^−/−^ and C57BL/6 mice were immunised intravenously (i.v) with 1×10^9^ pfu Ad-LacZ. (A) Histograms show representative CD73 staining on βgal_96_- (red) and βgal_497_- (blue) CD8^+^ T-cells at 14 and 85 days post immunisation in comparison to total CD8^+^ T-cells from naive mice. (B) CD73 staining on βgal_96_ and βgal_497_ CD8^+^ T-cells at 14 and 85 days post immunisation in comparison to total CD8^+^ T-cells from naive mice (n = 5, mean±SEM). (C) Time course showing specific CD8^+^ T-cells for βgal_96_- red/orange and βgal_497_- dark blue/light blue for C57BL/6 and CD73-/- mice respectively. Blood from 0, 14, 21, and 50 days post immunisation were stained with tetramers and analyzed by flow cytometry. Mean percentages of live tetramer-positive CD8^+^ lymphocytes are indicated (n = 5, mean±SEM).

Just as in MCMV infection, activated virus-specific CD8^+^ T-cells down-regulate the expression of CD73 (Figure 5 A/B), and at 85 days post infection virus-specific CD8^+^ T-cells re-expressed CD73 at levels similar to naïve CD8^+^ T-cells. However, compared to MCMV infection there was only a small difference between the inflating (βgal_96_) and non-inflating (βgal_497_) populations in CD73 expression at later time points. Therefore, to further assess the impact of CD73 deficiency in a second model, we infected both C57BL/6 and CD73^−/−^ mice with this Ad5-LacZ and tracked the epitope-specific CD8^+^ T-cell response longitudinally.

As with MCMV, we noted no difference in the frequency of response. In both C57BL/6 and CD73^−/−^ mice, βgal_96_-specific CD8^+^ T-cells gradually increased after infection, over the course of 50 days, while βgal_497_-specific CD8^+^ T-cells increased in frequency to 10% of total CD8^+^ T-cells at 14 days post infection and then gradually declined, being maintained thereafter at a low but stable frequency (Figure 5C).

## Discussion

Recently, CD73 has become recognised as an important mediator of anti-inflammatory responses and high CD73 expression on HIV specific CD8^+^ T-cells has been suggested as a hallmark of elite controllers [27]. To test the functional significance of CD73 in persistent infection, in the present study we investigated the effects of CD73 knockout on the evolution of CD8^+^ T-cells in mice infected with MCMV or replication-deficient adenovirus – two models where divergent CD8^+^ T-cell memory occurs. We show that the lack of expression of CD73 had no effect on the frequency of virus-specific CD8^+^ T-cells after MCMV infection or adenovirus immunisation and CD8^+^ T-cells showed the same proliferation kinetics, distribution, functionality and phenotype in both wild type C57BL/6 and CD73^−/−^ mice.

It is a feature of both the MCMV and adenovirus models that we can address the impact of CD73 on two quite distinct T-cell memory pools. The phenotype of M45-specific CD8^+^ T-cells is that of a central memory response, being CD27^Hi^, CD62L^Hi^, NKG2A^Lo^, CD127^Hi^, while the M38-specific CD8^+^ T-cells show an inflationary response and an effector-memory phenotype, CD27^Lo^, CD62L^Lo^, NKG2A^Hi^ and CD127^Lo^. It is of interest that there were no observable differences in viral specific CD8^+^ T-cells over the course of 100 days between C57BL/6 and CD73^−/−^ infected mice. This suggests that CD73 is dispensable to the formation of both a central and inflationary memory response. It will be nevertheless of interest to explore this further in models of T-cell exhaustion such as Lymphocytic Choriomeningitis Virus (LCMV), where immune regulation is potentially a more prominent key feature. Additionally, the function of CD39 (upstream of CD73) should be further addressed in such persistent virus infections, since this may provide a rate-limiting step. In addition to CD8^+^ T-cells, other cell types also express CD73, including endothelial cell populations. Of relevance, T-regs also express CD73 and produce adenosine which may, in an autocrine manner, inhibit their function [28], [29]. The lack of impact of CD73 depletion in both adenovirus and MCMV models suggests that this feature of T-reg biology is also not critical in moulding the evolving antiviral T-cell memory.

In conclusion, we show that lack of CD73 appears not to affect the production or maintenance of inflationary MCMV-specific or adenoviral vaccine-induced CD8^+^ T-cells, or the development of classical memory. The robust and reproducible nature of memory inflation in these models suggests it is closely regulated – however, alternative molecules must be sought to define the critical regulatory pathways *in vivo*. While we have shown a lack of impact in an otherwise physiologically intact mouse, it remains a possibility that in man, and especially in pathologic, dysregulated conditions such as CVID, the regulation of adenosine and the function of CD73 in this may play a more prominent role.

## Supporting Information

Figure S1 **Extracellular ATP metabolism.** ATP is dephosphorylated to ADP and to AMP by CD39. AMP is dephosphorylated to adenosine by CD73. ATP which binds to purinoceptors P2X and P2Y, is pro-inflammatory. By contrast, adenosine which binds purinoceptros of the P1 type, is anti-inflammatory.
(TIF)

Figure S2 **Phenotype of CD73^Hi^ and CD73^Lo^ populations of epitope-specific cells.** C57BL/6 mice were infected intravenously (i.v.) with 1x10^6^ pfu MCMV. (A) M38- and M45- specific CD8^+^ T-cells were gated on high (red) and low (blue) CD73 expression and CD27, CD127 and CD62L expression measured, shown are representative histograms for these staining's at 7 and 140 days post infection. (B) Levels of CD127 expression shown on CD73^Hi^ and CD73^Lo^ populations from CD8^+^ T-cells in naive mice and M38- and M45- specific CD8^+^ T-cells at 7 and 140 days post infection (n = 5, mean±SEM).
(TIF)
