# Correction: CD73 Is Dispensable for the Regulation of Inflationary CD8^+^ T-Cells after Murine Cytomegalovirus Infection and Adenovirus Immunisation

**DOI:** 10.1371/journal.pone.0121925

**Published:** 2015-03-25

**Authors:** The PLOS ONE Staff

Dr. Beatrice Bolinger is not included in the author byline. She should be listed as the third author, and her affiliation is 3: Department of Biomedicine, University of Basel, Hebelstrasse 20, 4056 Basel. The contributions of this author are as follows: Conceived and designed the experiments.

The correct citation is: Sims S, Colston J, Bolinger B, Emery V, Klenerman P (2014) CD73 Is Dispensable for the Regulation of Inflationary CD8^+^ T-Cells after Murine Cytomegalovirus Infection and Adenovirus Immunisation. PLoS ONE 9(12): e114323. doi:10.1371/journal.pone.0114323
