# Supplementary figures and images for: CD73 Is Dispensable for the Regulation of Inflationary CD8+ T-Cells after Murine Cytomegalovirus Infection and Adenovirus Immunisation
Source: PLoS One. 2014 Dec 9;9(12):e114323. doi: 10.1371/journal.pone.0114323 (PMC4260835; doi:10.1371/journal.pone.0114323)

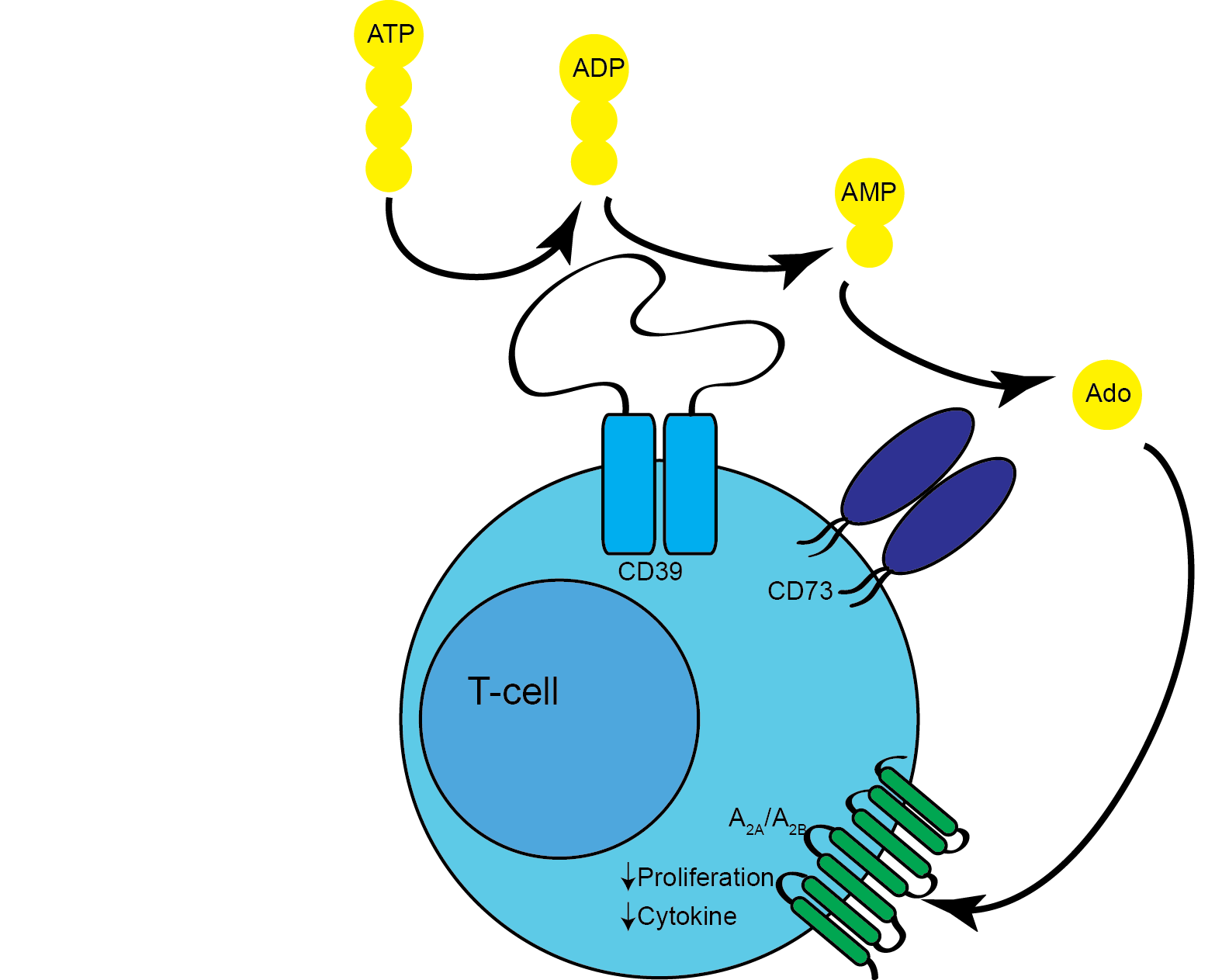

Supplement: Figure S1 — Extracellular ATP metabolism. ATP is dephosphorylated to ADP and to AMP by CD39. AMP is dephosphorylated to adenosine by CD73. ATP which binds to purinoceptors P2X and P2Y, is pro-inflammatory. By contrast, adenosine which binds purinoceptros of the P1 type, is anti-inflammatory. (TIF) [file pone.0114323.s001.tif]

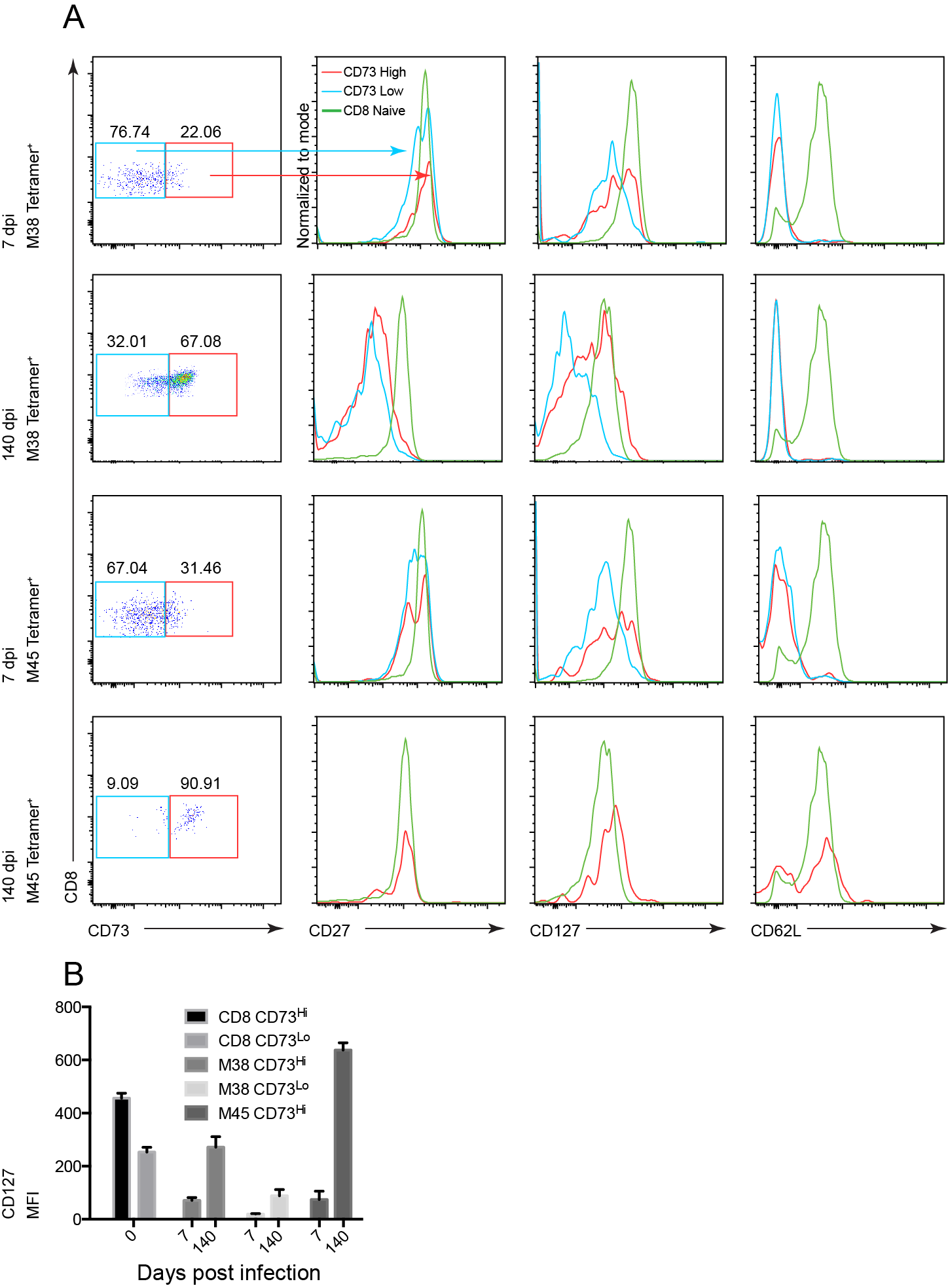

Supplement: Figure S2 — Phenotype of CD73Hi and CD73Lo populations of epitope-specific cells. C57BL/6 mice were infected intravenously (i.v.) with 1x10^6^ pfu MCMV. (A) M38- and M45- specific CD8+ T-cells were gated on high (red) and low (blue) CD73 expression and CD27, CD127 and CD62L expression measured, shown are representative histograms for these staining's at 7 and 140 days post infection. (B) Levels of CD127 expression shown on CD73Hi and CD73Lo populations from CD8+ T-cells in naive mice and M38- and M45- specific CD8+ T-cells at 7 and 140 days post infection (n = 5, mean±SEM). (TIF) [file pone.0114323.s002.tif]
